# A Wireless Data Acquisition System Based on MEMS Accelerometers for Operational Modal Analysis of Bridges

**DOI:** 10.3390/s24072121

**Published:** 2024-03-26

**Authors:** Hamed Hasani, Francesco Freddi, Riccardo Piazza, Fabio Ceruffi

**Affiliations:** 1 Department of Engineering and Architecture, University of Parma, 43121 Parma, Italy; hamed.hasani@unipr.it; 2 FIAMA S.r.l., R & D Department, 43126 Parma, Italy; riccardopiazza94@gmail.com (R.P.); fabio8320@gmail.com (F.C.)

**Keywords:** operational modal analysis, dynamic analysis, bridge structural health monitoring, wireless data acquisition system, modal identification techniques, MEMS accelerometers

## Abstract

This paper illustrates a novel and cost-effective wireless monitoring system specifically developed for operational modal analysis of bridges. The system employs battery-powered wireless sensors based on MEMS accelerometers that dynamically balance power consumption with high processing features and a low-power, low-cost Wi-Fi module that ensures operation for at least five years. The paper focuses on the system’s characteristics, stressing the challenges of wireless communication, such as data preprocessing, synchronization, system lifetime, and simple configurability, achieved through the integration of a user-friendly, web-based graphical user interface. The system’s performance is validated by a lateral excitation test of a model structure, employing dynamic identification techniques, further verified through FEM modeling. Later, a system composed of 30 sensors was installed on a concrete arch bridge for continuous OMA to assess its behavior. Furthermore, emphasizing its versatility and effectiveness, displacement is estimated by employing conventional and an alternative strategy based on the Kalman filter.

## 1. Introduction

Traditional methods for monitoring bridge health primarily depended on physical inspections, which come with inherent limitations. They are time-consuming, labor-intensive, and probably unreliable [1]. Furthermore, any delays in taking action or neglecting maintenance may lead to substantial future costs, especially for infrastructures with critical importance [2].

To overcome these drawbacks, techniques for bridge structural health monitoring (BSHM) have been developed, incorporating both static and dynamic approaches. Similar to static, dynamic analysis is divided into short- and long-term evaluations. The first one focuses on analyzing structural behavior during or after specific events, such as earthquakes, load testing of bridges, and responses to live loads. In contrast, the objective of the long-term assessment is to identify any deviations through the structural dynamic parameters over time, which would probably result in recognizing the presence, determining the severity and type, and pinpointing the location of damage or deterioration that may require maintenance or repair. This is achieved through a combination of data collection, analysis, and reporting [3].

Structural, environmental, and operational sensors serve different purposes in this process [4,5]. The structural sensors commonly used in BSHM include strain gauges [6], displacement transducers [7], velocity transducers [8], and accelerometers [9]. Among these, accelerometers play a key role in capturing the structural dynamic response, which are primarily categorized into three types: piezoresistive [10], piezoelectric [11], and microelectromechanical system (MEMS) [12,13]. With the advancements in MEMS accelerometers, their integration into BSHM has markedly increased. MEMS emerges as a practical alternative to other types, given its attributes such as low power consumption, high sensitivity, cost-effectiveness, and sufficient sampling frequency [4].

From another standpoint, sensing systems can be classified into wired and wireless. While the first employ physical cables for data transmission [14], wireless sensors utilize radio frequency or Bluetooth [15]. Despite offering significant advantages, such as ease of installation, relocation flexibility, remote monitoring, and cost-effectiveness, wireless sensing systems present distinct challenges. One of the primary concerns is their reliance on batteries as the primary power source. This constraint restricts the continuous operation of wireless sensors, limiting the duration of data collection and imposing limitations on their deployment in remote or inaccessible locations. Furthermore, wireless data transmission is inherently less reliable compared to its wired counterpart, often resulting in lower data transfer rates. Another challenge lies in synchronizing the data captured by multiple sensors. Unlike wired systems, where a central system clock housed at the data server provides a unified time reference, wireless setups face the complexity of coordinating data timestamps [16]. This issue can introduce errors in data interpretation and hinder the aggregation of sensor readings into meaningful insights. To address these probable transmission delays [17], various techniques have been developed, including time-stamp estimation and correlation functions. These methods aim to establish a consistent time reference across the network, enabling accurate synchronization and facilitating reliable data collection [4].

Assessing long-term behavior in bridges involves dynamic approaches to determine natural frequencies, mode shapes, and damping ratios [3]. Experimental modal analysis (EMA) and operational modal analysis (OMA) are commonly employed techniques. EMA uses controlled excitation sources for induced vibrations, while OMA relies on structural vibration during operational states, proving useful for continuous monitoring in huge and hard-to-access structures such as bridges and towers [4]. OMA offers economic advantages by eliminating the need for controlled excitation equipment. Common OMA methods include peak picking (PP), frequency domain decomposition (FDD), and enhanced frequency domain decomposition (EFDD) in frequency and stochastic subspace identification (SSI) in time domain analysis, offering two methods: SSI-data and SSI-covariance. To assess structural damage, damage-sensitive features (DSFs) are derived from modal identification techniques, relying on changes in dynamic responses. DSFs include shifts in natural frequencies, alterations in mode shapes, variations in strain levels, adjustments in damping properties, and modifications in energy dissipation [4]. Among these, mode shapes, less affected by environmental factors, offer valuable spatial information for damage localization.

These papers have significantly contributed to the progress of wireless monitoring systems for structures. Guérineau et al. [18] proposed a wireless SHM framework utilizing a seismic MEMS accelerometer for bridge monitoring under ambient excitation. In parallel, Araujo et al. [19] worked on a wireless sensing system with high synchronization for OMA, which was developed under a Spanish ministry-funded project. He et al. [20], Hu et al. [21], and Whelan et al. [22] independently explored vibration-based monitoring on a highway bridge using wireless measurements. Komarizadehasl et al. [23] investigated a low-cost triaxial accelerometer based on Arduino technology and applied it in the eigenfrequency analysis of a footbridge. Reviews by Abdulkarem et al. [24], Zhou and Yi [25], Lynch [26], and Sabato et al. [27] underscore the growing importance of wireless sensor networks in structural health monitoring. Kim et al. [28] applied a wireless sensing system on the Yeondae Bridge in Korea. Lynch et al. [29] compared a low-cost wireless sensor network in the Geumdang Bridge, Korea, to a commercial system, showing comparable results. Dai et al. [30] designed, implemented, and tested a wireless sensor network (WSN) for BSHM on the ZhengDian viaduct bridge in China. Reyer et al. [31] proposed a wireless sensor network using readily available sensors for capturing and analyzing vibration features of bridges. Gutiérrez and Garita [32] developed a cost-effective wireless bridge monitoring system prototype with a web interface for vibration data analysis. Asadollahi et al. [33] deployed a wireless smart sensor network on a cable-stayed bridge in South Korea, collecting extensive data for statistical analysis of modal properties and exploring temperature–natural frequency relationships. Furthermore, Chae et al. [34] proposed a wireless sensor system for BSHM using ZigBee for short-distance and code division multiple access (CDMA) for long-distance communication, integrating a versatile one-channel data logger. The collection of these papers demonstrates the extensive range of applications and innovative advancements in wireless monitoring systems, which serve to improve bridge structural health assessment.

On the other hand, evaluating the displacement response of the bridge, including each member, is necessary to detect fatigue damage and preempt brittle fracture. It is important to specify displacement responses in members that are susceptible to stress concentrations. Accurate assessment of displacement response under live loads, a dominant factor in fatigue damage, is vital for effective bridge maintenance [35]. Converting acceleration to displacement allows for determining the bridge’s movement in response to external forces. While acceleration reveals dynamic forces, displacement response provides crucial information about the actual deformation and movement of bridge elements.

Amidst the challenges faced by wireless monitoring systems, which often engage with issues related to system lifetime, hardware affordability, system configurability, and data reliability, this study illustrates an innovative and cost-effective wireless data acquisition system designed for OMA in bridges to address these concerns. The system comprises a series of custom-designed, battery-powered wireless sensors equipped with tri-axial MEMS accelerometers (ADXL355 version, from Analog Devices Inc., Cambridge, MA, USA), a cost-effective Wi-Fi module, a commercial Wi-Fi 4G LTE router powered by a solar panel, and a web-based graphical interface to configure the test and receive, store, and download data from the field. To optimize energy consumption, the system employs low-power strategies by using energy-efficient hardware components and a standby mode to minimize continuous power usage. The gateway, along with the solar panel utilized in the bridge monitoring, boasts versatile functionality, suitable for various off-grid applications requiring 12 V batteries. It includes a long-lasting lithium battery capable of over 3000 cycles, significantly surpassing lead-acid batteries. The 25-watt waterproof panel is durable and equipped with a user-friendly connector for easy plug-and-play. The paper also addresses various challenges inherent in wireless communication, including data preprocessing, synchronization, system lifespan, and ease of remote configuration.

This paper initially details the hardware design of the sensor in Section 2.1.1, emphasizing the participation of the components such as MEMS accelerometer, wireless module, memory, host microcontroller, USB communication, battery, antenna, and enclosure box on the printed circuit board (PCB). Then, the workflow and the operational process upon sensor awakening are elaborated in Section 2.1.2. The involvement of the other two physical components of the wireless system—router and cloud platform—is discussed in the subsequent sections (Section 2.2 and Section 2.3). Section 3 covers the pre-analytical process involved in signal processing of the monitoring system, focusing on sensor calibration, and data preprocessing steps for eliminating the undesired frequencies and implementing the synchronization technique to multiple wireless sensors. A laboratory test is then conducted (Section 4.3) to validate the functionality and precision of the monitoring system, using a 4-story shear-type structure on a shaking table, before its real-world application. After preprocessing steps, such as filtering and synchronization, its modal parameters are extracted using frequency and time domain modal identification methods. Additionally, a computational model is employed to validate the experimental results with finite element modeling (FEM). Following the verification, in Section 4.4, a system composed of 30 wireless sensors is installed on a concrete arch bridge for continuous OMA, and the dynamic results are reported. Highlighting the system’s adaptability and efficiency, displacement is determined from the acceleration data using conventional methods during laboratory testing and an alternative approach based on the Kalman filter for the real-world case study.

**Figure 1:**
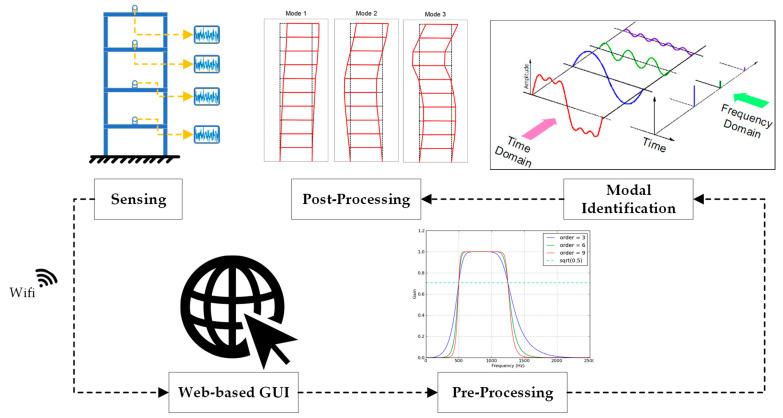
The workflow of the monitoring system.

The procedure for assessing the dynamic behavior of a structure using a wireless monitoring system is illustrated in Figure 1. The process initiates with data acquisition, progressing through the pre-processing stage to appropriately prepare the data for subsequent analysis. It then advances to the modal identification, with the objective of extracting the dynamic characteristics inherent to the structure. The procedure concludes with the post-processing and damage detection step. In these sequential stages, the data acquisition system (DAQ) component assumes a crucial role in detecting the targeted vibrations emanating from the structure. The precision of the DAQ holds utmost significance, as it directly impacts the accuracy of the ensuing data analysis.

## 2. Monitoring System Overviews

**Figure 2:**
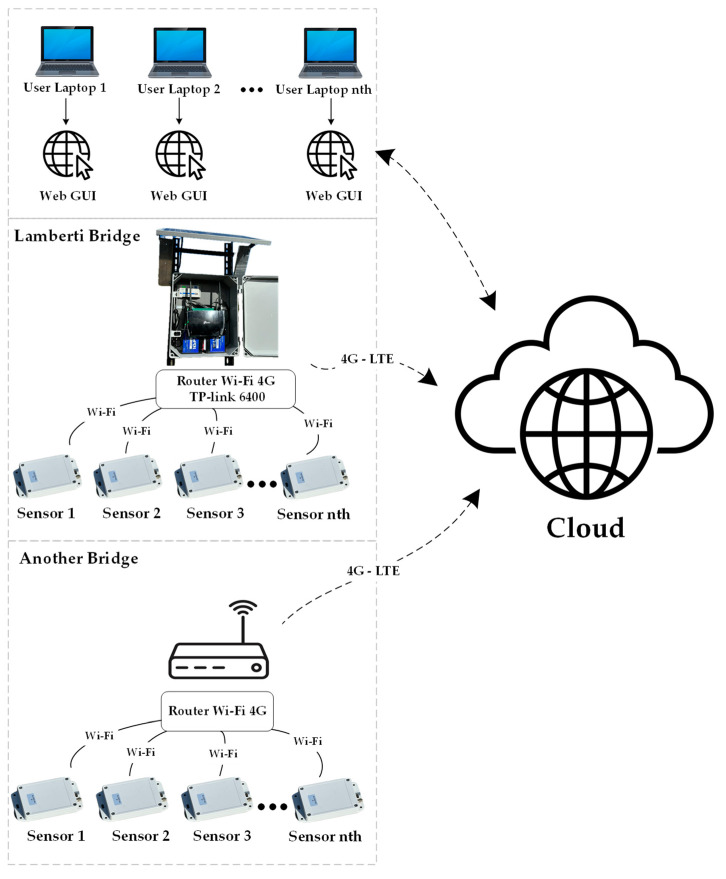
An overview of the system’s operation.

Figure 2 illustrates the primary components of the system and delineates their interconnections. As depicted, the cloud platform acts as an intermediary connection between the sensing system, comprised of sensors mounted on the bridge and routers, and the user configuration that sets the test parameters through the web-based GUI. This system can be simultaneously installed on multiple bridges, receiving structural vibrations from numerous wireless sensors and facilitating the storage and transmission of data for subsequent analytical processes.

The sensor serves as the core element of this system, and it is essential that it satisfies all the prerequisites for prolonged remote monitoring [36]:

1. Easy configuration is essential and should be accomplished remotely.
2. Users should have the capability to configure all sampling parameters, including frequency rate, duration, and starting time.
3. It should have the capability to sample continuously for a minimum of one hour at a rate of 125 Hz, which is the amount of data typically required for OMA applications.
4. Accelerometer measurements must meet specified criteria for precision, repeatability, and noise levels. This includes ensuring that the frequency range falls within the minimum required frequency and the maximum measurement frequency, avoiding saturation of the instrument, maintaining a minimum signal-to-noise ratio (≥10 dB), and ensuring adequate sensitivity for A/D conversion.
5. Data synchronization for modal analysis must be precise: The synchronization error is advisable to be less than 1/10 of the sampling period.
6. Connection must be wireless, stable, and reliable.
7. It must be reliable and able to operate without external maintenance throughout its life cycle.
8. It must be able to work outdoors; all of its components must function at all temperatures, and the enclosure must be weather-resistant.
9. The cost of each sensor should be affordable, and the components utilized should be readily accessible.
10. To ensure the transparency of the analysis process, it is imperative that data be transmitted and made fully available.

The subsequent physical component pertains to the router, a crucial element responsible for rerouting the localized Wi-Fi network emanating from the sensors to the web server via its 4G LTE connection. Its power source can be derived either from the electrical grid or from a solar panel coupled with a battery system. The ultimate component of the system is the cloud platform, encompassing a backend server responsible for receiving messages directly from field sensors and systematically storing the acquired data. The backend module is additionally tasked with overseeing device configurations and managing user access. Complementary to this is the frontend component, primarily constituted by a graphical user interface. This interface facilitates the retrieval of comprehensive information concerning the sensors, enables the examination of their operational status, and supports the downloading of data in various formats. The following sections provide comprehensive details of the individual components comprising the system.

### 2.1. Sensor

#### 2.1.1. Hardware

**Figure 3:**
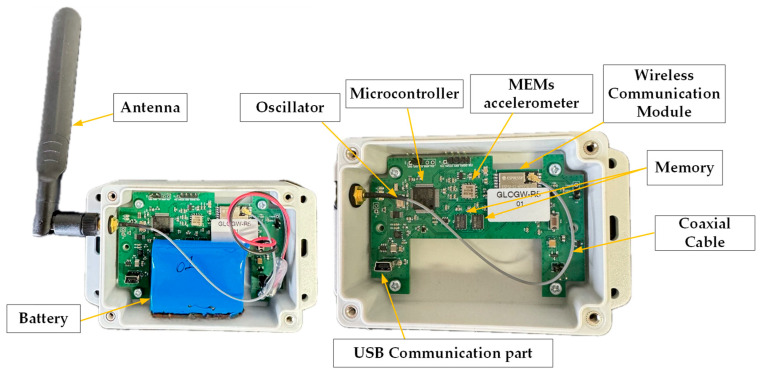
100×70 (mm) PCB equipped with MEMS accelerometer.

The sensor is comprised of commercially selected components to meet the specifications of this application. A custom-designed PCB integrates the accelerometer, memory, microcontroller, network module, and battery. Each of the mentioned components is linked to the host microcontroller for operational management and is connected to the batteries as their power source. More in-depth information regarding the functional aspects of each primary constituent part of the PCBs is elaborated in the subsequent sections. Figure 3 visually depicts the designed PCB.

##### Accelerometer

For the selection of the accelerometer’s type, a compromise has been made between the required performance, power consumption, footprint size, and price. This requirement has guided the choice towards a tri-axial MEMS-based accelerometer with digital output, the ADXL355 from Analog Devices Inc. (Cambridge, MA, USA).

**Figure 4:**
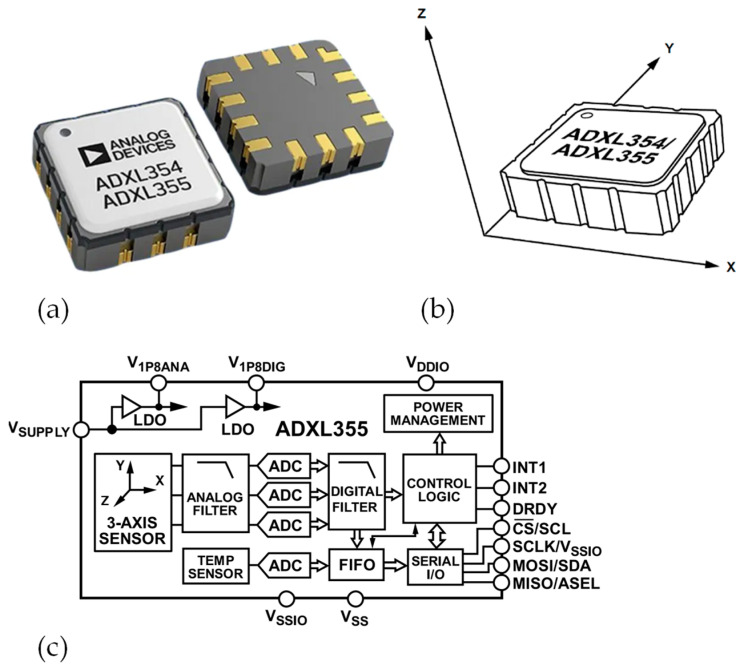
The employed MEMS accelerometer from ADXL 355 version: (**a**) picture, (**b**) directions, and (**c**) functional block diagram.

It consists of three sets of MEMS sensing elements that follow separate differential signal trajectories to reduce noise and drift. Filters are applied both before and after these ADCs to reduce noise. The range, output data rates, and filter corner frequencies can be customized through register access. In this paper, a range of ±2 g was incorporated, and the frequency rate of the sensors was adjusted within the range of 62.5 Hz to 500 Hz for both experimental and real-case scenarios. To generate the sampling frequency, an internal clock was chosen which has a device-to-device deviation of 1.4%, along with a temperature-dependent variation of 1.2%. Therefore, this needs to be taken into account in the post-processing phase, as it will require compensation with ad hoc algorithms (Section 3.1). The utilized MEMS accelerometer, ADXL355 version, is illustrated in Figure 4, and Table 1 reports its key parameters under these conditions: zero *x*-axis and *y*-axis accelerations, and *z*-axis acceleration of 1 g, alongside an output data rate (ODR) of 500 Hz.

##### Wireless Module

For this study, the Wi-Fi wireless communication standard is selected due to its high throughput, network simplicity, and suitable coverage range of around 200 m outdoors. The onboard Wi-Fi communication is managed through the Espressif ESP32 module from Espressif Systems company (CHN, CZE, IND, SGP, BRA), which comprises a dual-core microcontroller. One core is dedicated to network protocol management, while the other is devoted to the user application. In this setup, the ESP32’s task involves reading data from memory, sending it via Wi-Fi to the cloud, and receiving functional configurations from the cloud. Additionally, it handles keeping the time synchronized with the network, leveraging the time synchronization function.

Synchronization

To achieve sensor synchronization within the wireless network, the IEEE 802.11 timing synchronization function (TSF) is used. The TSF, a distinct timer maintained by the network access point (the router), is disseminated to all devices within its network. This approach ensures the synchronization of timers across all stations within the same basic service set (BSS). The more usual approach of retrieving absolute time from an NTP server can usually maintain time to within tens of milliseconds over the public Internet, but asymmetric routes and network congestion can cause errors of 100 ms or more. Both methods (NTP and TSF) were utilized to enhance synchronization management. It is important to link each sample to a numerical value that can be traced back to absolute time; experimentally, the TSF-based methodology is more precise for our specific objective. Through this method, a synchronization error noticeably lower than the sampling time (~2 ms) is attained. During laboratory tests, data were sampled between 62.5 and 500 Hz; while in the bridge case study, the sampling rates were set at 62.5 and 125 Hz. As validated through various experimental test setups, this timing error was found to be inconsequential to the precision of the results, causing no significant complications in the synchronization of our collected data. The synchronization process employed in this study is further explained in Section 3.1.

##### Memory

To store data, two 8 Mbyte ultra-low power flash memory modules have been selected. Each data sample is 16 bytes in size (3 bytes per axis, 4 bytes for timestamp in microseconds, 2 for error detection, and 1 byte for memory alignment), enabling the storage of up to two hours’ worth of sampling at 125 Hz, meeting one of the system requirements. The fundamental aspect of these components is their ultralow-power characteristic. Since they need to remain powered on for up to two hours, it is crucial that they have extremely low energy consumption, both when idle and during the read and write phases. Thus, it should not come as a surprise that these components have a limited storage capacity. Flash memories available in the market can store hundreds of times more data in the same physical size. However, the selection of these components was not primarily guided by the goal of maximizing storage capacity.

##### Host Microcontroller

As previously stated, the primary element crucial for the board’s operation is the host microcontroller, tasked with executing the following functions:

- Configure the accelerometer and read data.
- Configure the memory, read, and store data.
- Configure the communication module.
- Receive operational configuration from the network and apply it correctly.
- Handle external USB communication.
- Enable the entire board’s power-saving mode, activating other components only when necessary.
- Synchronize and maintain the board’s time base (clock).

For these purposes, the STM32L4 line of microcontrollers has been selected. They offer dynamic voltage scaling to balance power consumption with processing demand, low-power peripherals (LP UART, LP timers) available in stop mode, safety and security features, as well as smart and numerous peripherals. Additionally, they feature advanced and low-power analog peripherals. These features enable the microcontroller to consume less than 8 uW in stop mode—the mode the board must maintain when not actively sampling accelerometer data. Serving as the timing base for the board, the microcontroller is equipped with two oscillators: one operating at a frequency of 32 kHz to uphold the real-time clock, and a second oscillator at 8 MHz necessary for the dedicated synchronization timer.

##### USB Communication

A USB communication protocol has been integrated to establish a connection between the device and a computer, serving the following purposes:

- Configuring Wi-Fi network parameters (SSID and password).
- Performing tests to assess the health of the components.
- Downloading data in the event of unavailability of the Wi-Fi network.
- Updating the device firmware.

USB communication within the board is entirely managed by the host microcontroller, while on the PC side, a compact C++ application has been designed to execute all the mentioned operations.

##### Battery

The choice of the battery was influenced by three primary requirements:

- Minimum five-year lifespan: Ensuring a lasting operational period is essential, given that the sensors may be deployed in hard-to-reach and challenging environments. They must supply ample energy to support prolonged monitoring over an extended period.
- 30 min of daily operation: The battery’s lifespan is contingent on the volume of data to be collected. A battery supporting 20 Wi-Fi connections per day will undergo more strain compared to one facilitating a connection once a week.
- 400 mA pulsed capacity: The battery should have the capability to deliver up to 400 mA, which represents the maximum energy demand during a Wi-Fi connection.

The consumption profile of the sensor during a standard operational day is depicted in Table 2. As represented, it can be divided into three reported segments.

To satisfy the demand, an 8500 mAh thionyl chloride ($SO{Cl}_{2}$) battery was chosen. Thionyl chloride is utilized as the positive electrode (anode) in certain lithium batteries, with lithium constituting the negative electrode (cathode). These batteries, although non-rechargeable, are characterized by numerous advantages, such as high energy density, a wide range of operating temperatures, and a longer storage and operational life when compared to other types of lithium batteries. As discovered during laboratory testing, the main disadvantage of thionyl chloride batteries is their diminished capability to deliver high pulsed currents. Consequently, a two-battery pack was configured by incorporating a second (rechargeable) lithium (Li) battery. While the five-year lifetime of the product is assured by the primary $SO{Cl}_{2}$ capacitor, the handling of consumption peaks in Wi-Fi communication is facilitated by the lithium component.

##### Antenna

The importance of antenna selection is underscored when the sensor is situated at considerable distances from the wireless access point (e.g., on a 300 m bridge where the access point is centrally positioned). In such cases, the use of high-gain antennas becomes necessary. Moreover, the sensor might be positioned in areas obstructed by obstacles like guardrails, walls, or barriers. Consequently, an omnidirectional high-gain antenna (8 dBi) was opted for, capable of being connected to the sensor box either directly or through a 1-m extension cable, depending on the specific requirements. The option with an extension cable also includes a magnetic base for convenient placement on metallic objects.

##### Enclosure Box

The sensor’s concluding hardware element is its casing, designed to mitigate the impact of external environmental factors on the sensors. A selected weather-resistant enclosure possesses an IP67 protection rating and features mounting holes, with 10.5 cm as the length, 7.5 cm as width, and 4.0 cm as height.

**Figure 5:**
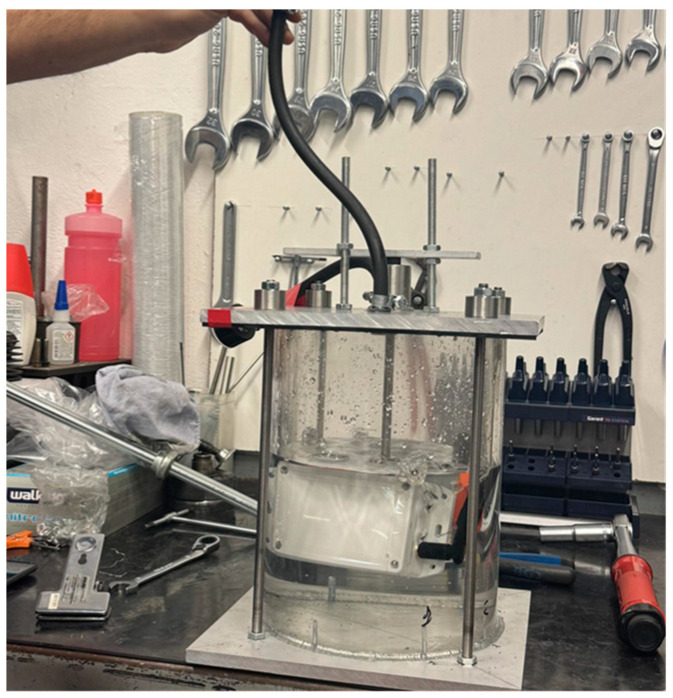
Experiment to assess protective enclosures for shielding against water ingress.

As depicted in Figure 5, an experimental investigation was conducted with the aim of assessing the efficacy of the protective enclosures in fully isolating the PCBs from external environmental influences. To achieve this objective, the encased PCBs were submerged within a cylindrical vessel filled with water. Subsequent to immersion, the vessel was securely sealed, and air was introduced to elevate internal pressure. The findings unequivocally demonstrate the effectiveness of the protective enclosures in safeguarding the PCBs against water ingress, thereby affirming their capability to maintain isolation and shielding from external environments.

#### 2.1.2. Workflow

**Figure 6:**
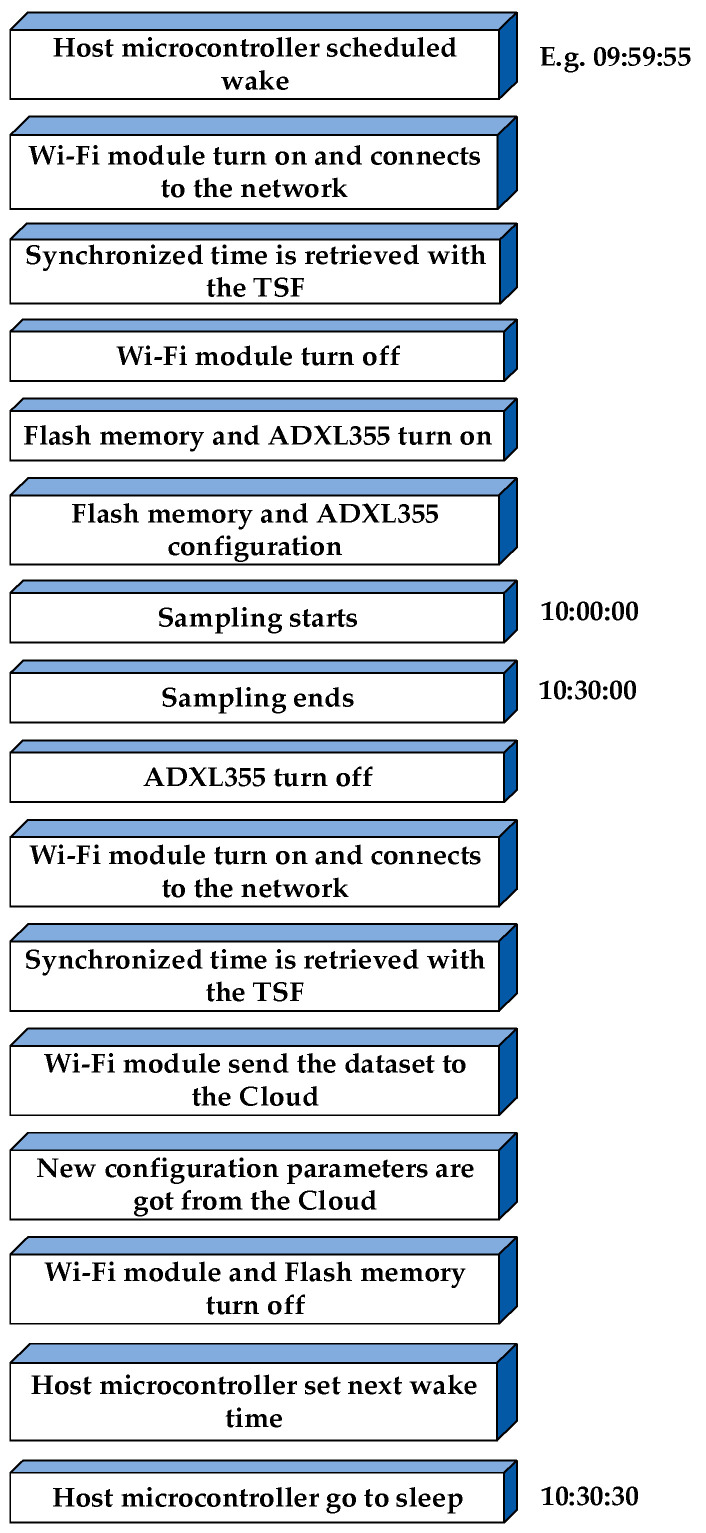
Operational process upon sensor awakening.

In this section, the operational process of the sensor is briefly described. As previously mentioned, it is active only for a small part of the day, with the remaining time spent in energy-saving mode. When energy-saving mode is engaged, the sensor is rendered completely inactive and remains inaccessible from the outside, except through a USB connection. The sequence of operations initiated when the sensor is “awakened” is depicted in Figure 6; the time of day at which this awakening occurs and the subsequent operations are entirely configurable by the user.

As illustrated in Figure 6, the fundamental goal of the entire process is to conserve battery power through the implementation of a strategy involving the activation and deactivation of all components. This method of managing power consumption distributes the energy among multiple sources or devices. This feature is particularly important for electronic devices with onboard batteries, which require significant amounts of power to operate. The devices are selected based on usage and only powered when necessary, through specific circuit breaker separators. The effects are that energy consumption is then directed to the active part during operation. The workflow commences with the activation of the host microcontroller. Subsequently, the Wi-Fi module is triggered to engage the time synchronization function, aligning the board time with the base station time. The internal timer, powered by the 8 MHz crystal, is responsible for maintaining the board time. Following this, the Wi-Fi module undergoes deactivation, whereas the flash memories and the ADXL355 are activated and configured. After a temperature measure, the sampling period begins as the host microcontroller initiates the reading of data from the ADXL355, subsequently storing it in the flash memory. Each recorded data point encompasses a quadruple: timestamp, acceleration in the *x*-, *y*-, and *z*-axis. After concluding the sampling phase, the second temperature measure is performed and then the Wi-Fi module reactivates. Initially, the time synchronization function is utilized to realign the board time with the network time, allowing for the management of any drifts or errors in the measured time. Subsequently, the Wi-Fi module transmits all the gathered data to the cloud. Upon the completion of these operations, the host microcontroller powers off every other component, establishes its wake time, and engages its ultra-low power mode.

### 2.2. Router

The second physical component of the system is the router, tasked with transmitting data received from the sensors via Wi-Fi to the cloud. For global network transmission, it relies on a 4G internet connection. To manage costs efficiently, the TP-Link 6400, a commonly adopted standard device for this application, has been selected. However, it should be noted that all the requested tasks could have been performed by any commercially available 4G router. This device features a SIM card slot, enabling it to establish a wireless internet connection. The router also requires a reliable power source. During internal functional tests, it was directly connected to the electrical grid using its power adapter. However, for external use, a configuration involves utilizing a pack containing two 96 Wh lithium-ion batteries. These batteries, along with a dedicated power supply, are connected to a 25 W solar panel. All components used are commercially available and can be easily found in any electronics store.

### 2.3. Cloud Platform

**Figure 7:**
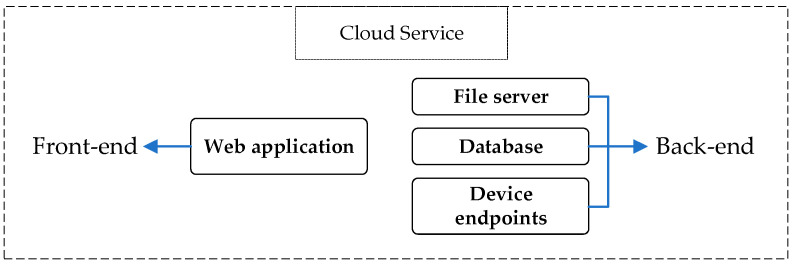
Cloud service division.

The last element of the monitoring system is the cloud, comprising a web application developed on PythonAnywhere. It is an online integrated development environment (IDE) and web hosting service, operating as a platform as a service (PaaS), built based on the Python programming language. Figure 7 illustrates the division of the cloud service in this study. The terms “front-end” and “back-end” in computer science are used to respectively indicate the part of a program that is visible to the user and with which the user can interact—usually a user interface—and the part responsible for the actual functioning of these interactions.

In our case, the back-end consists of the following:

- Device endpoints: These serve as the “addresses” to which the sensors in the field connect for sending data and receiving configuration.
- File server: This is a memory block where data received from devices are stored and sorted by device (using the unique MAC address of the devices as a reference) and timestamp. All data are stored in a super-compressed format to save disk space and are converted into a human-readable format when downloaded. For instance, a 60-min sampling at 125 Hz consists of a 7-megabyte file in compressed format and a 17-megabyte CSV format file.
- Database: This is a structured query language (SQL) database, which consists of highly structured tables. Each row represents a data entity, and each column defines a specific information field. In our case, it is responsible for storing the lists of users, their associated devices, their events, and their functional configurations.

**Figure 8:**
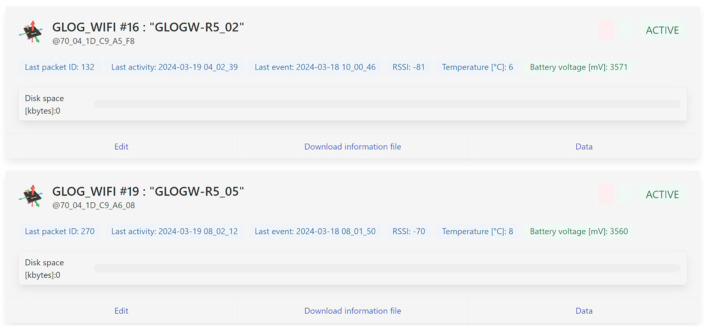
An excerpt from the device page, which shows two active devices with their last activity.

**Figure 9:**
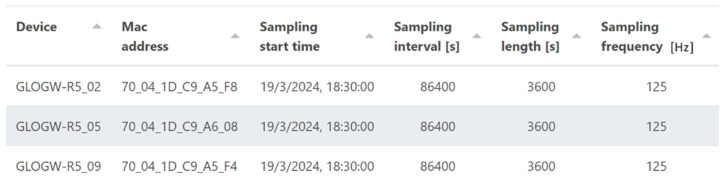
Configuration page for each test setup.

**Figure 10:**
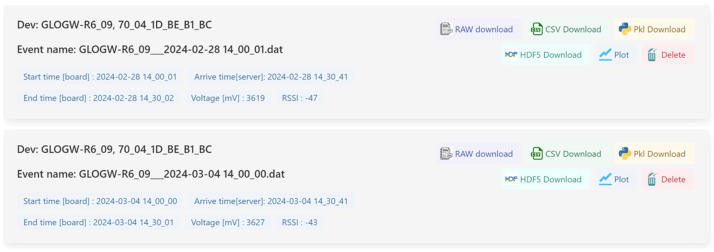
File page.

The front-end instead consists of a simple GUI with four screens:

- A page dedicated to user authentication and registration;
- A device page (depicted by Figure 8) that showcases a list of devices linked to each user, presenting essential details, such as the last connection time, last sampling time, last measured temperature, and battery status;
- A configuration page (Figure 9) featuring a table for device configuration. The table is structured in rows, where each row corresponds to a device uniquely identified by a user-set name and its MAC address. Each device can be configured with the following:
  - Sampling start time: exact time at which the device should start sampling;
  - Sampling interval: time between each sampling, in seconds;
  - Sampling period: desired duration of the sampling, in seconds;
  - Sampling frequency: frequency at which the accelerometer should operate, in Hz;
  - Get config interval: the maximum time between two connections to the server. If set lower than the sampling interval, it allows the sensor to connect to the server to download the configuration without performing the sampling part. This feature allows the system to be more “responsive,” albeit at the expense of battery life.
- A file page (illustrated by Figure 10) presents the list of data received from a particular device. Each dataset includes information about the start and end times of the sampling process. Users have the option to download the data in either CSV or HDF5 format.

## 3. Data Pre-Analytical Process

Pre-processing of the data before the implementation of modal identification algorithms stands as an important step in ensuring result accuracy, wherein various methods are employed to eliminate noise and artifacts from the measured signals. To minimize the influence of noise and undesired frequencies, filtering methods such as low-, high-, or band-pass are employed. These techniques serve to improve the signal-to-noise ratio by focusing on enhancing the specific frequency range of interest. Moreover, the detrending filter is employed to eliminate the influence of low-frequency trends or drifts present in the data. Additionally, external interference and measurement errors may introduce artifacts during data acquisition. To address these challenges, techniques such as signal interpolation, outlier detection, and data smoothing are utilized. In addition to the previously addressed considerations for the pre-processing step, achieving synchronization among the recorded data by multiple sensors is crucial to increase the precision in the extracted dynamic parameters, such as mode shapes of the structure [38]. Owing to the direct physical connection of wired sensors to a centralized data acquisition system through cables, these sensors can be controlled directly by the system, guaranteeing precise timing and synchronization in data acquisition. Conversely, due to the intrinsic characteristics of wireless connections, achieving synchronization is inherently intricate. Techniques such as time-stamp estimation and cross-correlation functions are employed in wireless sensor networks to evaluate signal propagation and compensate for transmission delays. Section 3.1 outlines the employed data synchronization process for the acceleration data recorded by the proposed wireless sensing system.

### 3.1. Synchronization Process

Because the sensing system operates in a wireless network and deals with the recorded data from multiple sensors, synchronization issues arose, as outlined below:

- The commencement times exhibited occasional slight variations.
- The sampling frequency occasionally deviated slightly (e.g., a board sampled at 125.8 Hz instead of 125 Hz).

**Figure 11:**
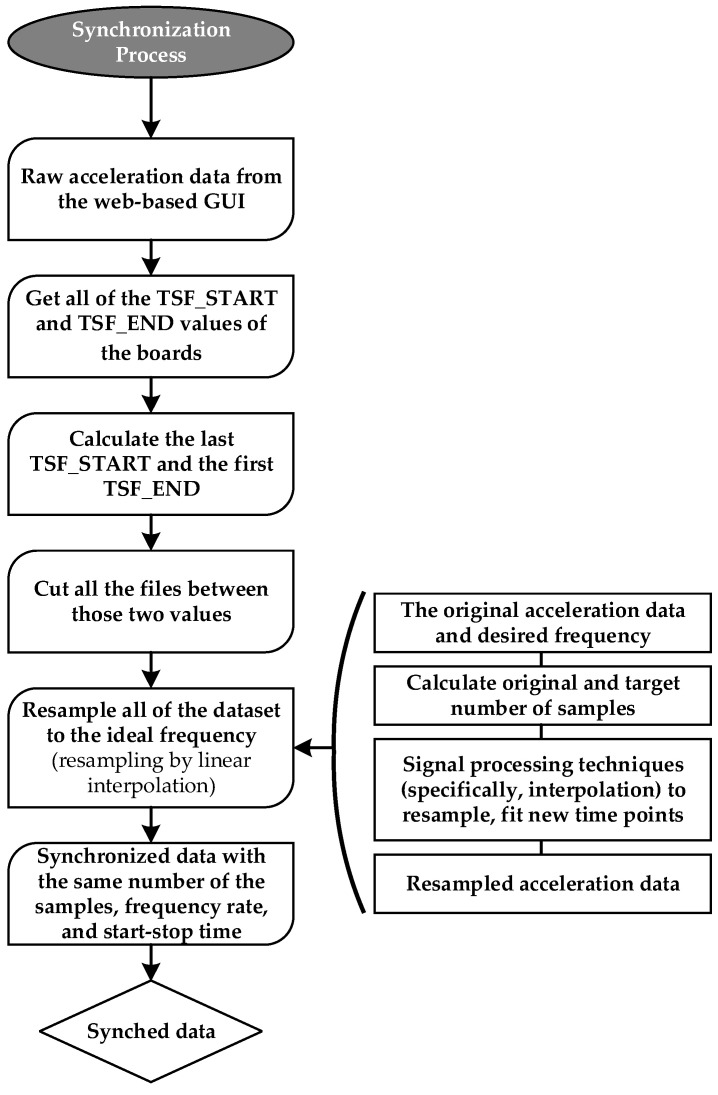
Data synchronization steps for the proposed wireless system.

Figure 11 depicts a flowchart outlining the employed data synchronization process within the system. Upon acquiring raw acceleration data from all sensors, TSF-START and TSF-END values are determined for each sensor via Wi-Fi protocol. Subsequently, the acceleration data from each sensor are segmented based on these determined start and end times. It is noteworthy, as discussed in Section Synchronization, that the synchronization achieved through TSF is expected to result in an error below the sampling time of approximately 2 milliseconds. Consequently, there exists the potential for a one-sample shift error in the recorded data from multiple wireless sensors, sampled at rates exceeding 500 Hz. This concern arises because, in scenarios with a frequency rate above 500 Hz, each sample is recorded with a time interval of less than 0.002 s. This may raise concerns regarding high-precision synchronization in the system. However, in this study, the selected sampling rates for the bridge OMA are within the range of 62.5 to 125 Hz (Section 4.2), and for the laboratory testing, 62.5, 125, and 250 Hz (Section 4.1). With the anticipated synchronization error of less than 0.002 s for the system, there is no synchronization concern for data captured at these frequency rates, with the time increments of 0.016, 0.008, and 0.00390625 s, respectively. Additionally, to compensate for differences in the sampling frequency among various sensors (Section Accelerometer), signals are resampled at the ideal sampling frequency using a common timestamp and resampling by linear interpolation. Consequently, precise synchronization is ensured by determining the exact start and stop times alongside the uniform sampling rate.

## 4. Experimental Process

### 4.1. Sensor Noise Characterization

This section investigates the noise characteristics of the sensing system across three axes, X, Y, and Z, through a controlled setup to isolate intrinsic noise properties, measuring peak-to-peak noise, root mean square (RMS) noise, and noise density (in μg/$\sqrt{Hz}$) along each axis. In accordance with Section Accelerometer, documented from the manufacturer’s data sheet [37], the reported noise density of the MEMS ADXL 355 version, operating under 25 °C temperature conditions, is documented as 22.5 μg/$\sqrt{Hz}$, with specific parameters set as follows: zero *x*-axis and *y*-axis accelerations, and *z*-axis acceleration of 1 g, alongside an ODR of 500 Hz.

**Figure 12:**
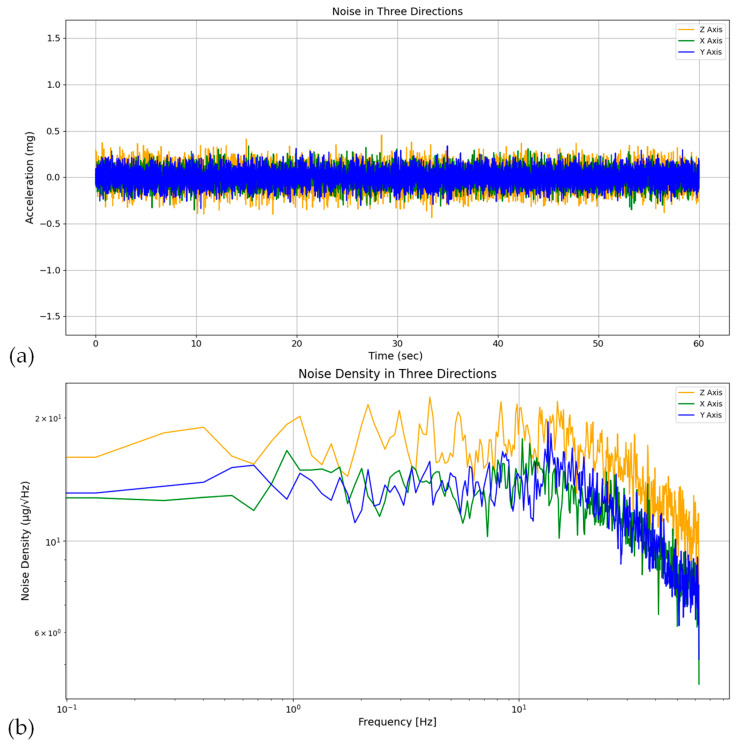
Comparison of the recorded noise in three directions: (**a**) in the time domain and (**b**) in the frequency domain.

In order to verify the noise characteristics of the proposed system in accordance with the manufacturer’s specifications, the system underwent static conditions. During this phase, data were recorded along the *X*, *Y*, and *Z* axes under consistent environmental and experimental conditions, specifically at a temperature of 25 °C and an ODR of 500 Hz. These recordings show the inherent noise characteristics of the sensor. Subsequently, the noise density plot in the Z direction was computed, revealing a value of 22.5 μg/$\sqrt{Hz}$, consistent with the manufacturer’s provided data. However, to ensure equivalence between noise characterization and both laboratory test conditions and real-world bridge application scenarios as delineated within this study, Figure 12a,b were presented. These figures illustrate the intrinsic noise characteristics under an ODR of 125 Hz along the *X*, *Y*, and *Z* axes, showcased in both the time and frequency domains, respectively. Complementing this analysis, Table 3 presents a comprehensive summary of sensor noise attributes across the three axes, as determined through the experimental protocol outlined above.

It is worth mentioning that, as depicted in Figure 12b, the noise density representation (in μg/$\sqrt{Hz}$) for each axis is derived through the computation of the power spectral density (PSD) plot ($g^{2}/Hz$) of the acceleration dataset (measured in g) within a static setting (termed as noise data), employing Welch’s method. To obtain the noise density values in μg/$\sqrt{Hz}$, the square root of the dataset is computed, subsequently multiplied by $10^{6}$.

As reported in the table, the measured data are consistent with the specifications of the chosen accelerometer.

### 4.2. Preprocessing Step

**Figure 13:**
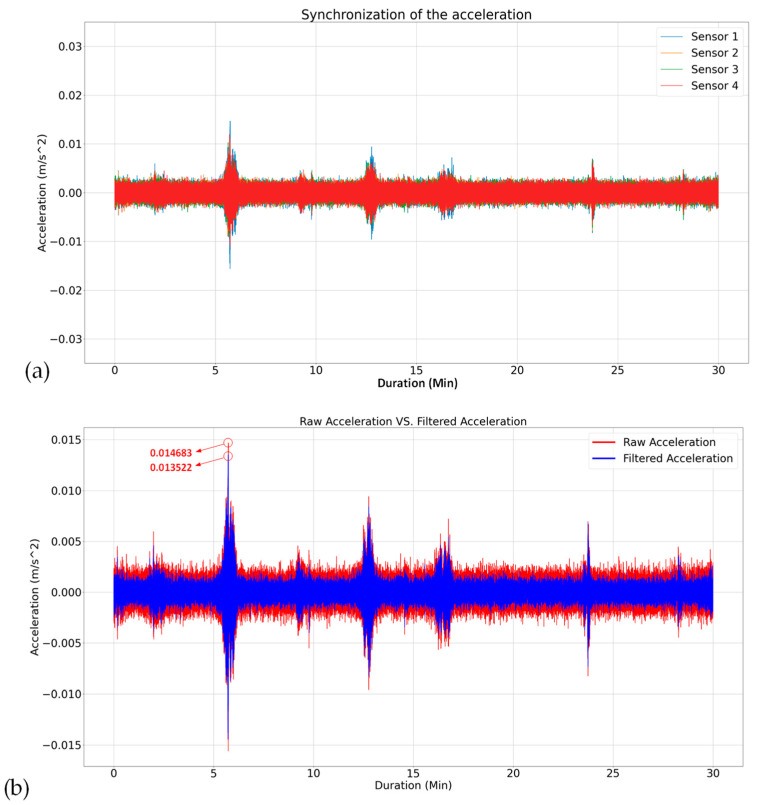

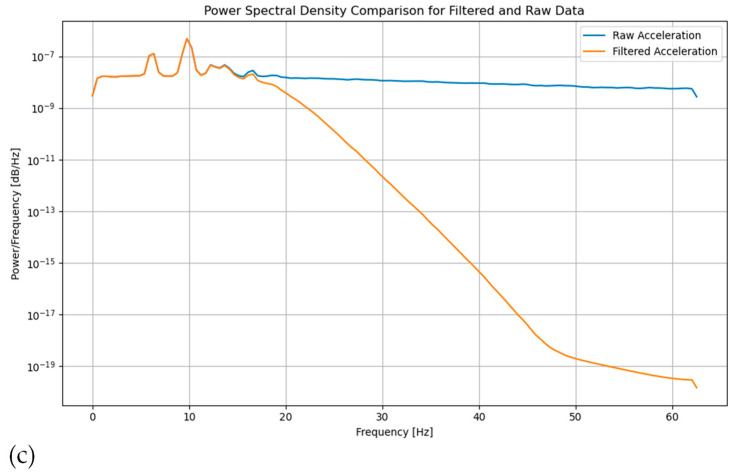
Application of the pre-processing step: (**a**) after detrending and synchronizing data from four accelerometers, (**b**) following the implementation of low-pass filtering, and (**c**) a comparison between the PSD plot of the acceleration data from one sensor in one direction before and after pre-processing.

The raw acceleration data recorded by the accelerometers underwent preprocessing procedures as mentioned in Section 3. As a result, Figure 13a visually represents the synchronized data acquired from one of the conducted tests, and Figure 13b,c illustrate the data comparison before and after filtering in the time and frequency domains, respectively. In this particular test configuration, a low-pass filter, Butterworth type, with a cutoff frequency of 20 Hz and an order of 2 was employed, along with the integration of detrending filtering. As depicted in Figure 13b, following the application of the filter, the peak value in the time domain plot reduced from 0.014682 $m/s^{2}$ to 0.013522 $m/s^{2}$ and a decrease in the amplitude of frequency components beyond 20 Hz is obvious in the frequency domain (Figure 13c). In reference to Figure 13c, it is pertinent to note that the input acceleration data were considered in $m/s^{2}$, and the analysis utilized Welch’s method for the computation of the PSD. To enhance clarity and facilitate comparative assessment across frequency domains, the PSD was depicted in decibels per Hertz (dB/Hz), thereby employing a logarithmic scale for the representation of power values with respect to frequency.

### 4.3. Laboratory Validation

**Figure 14:**
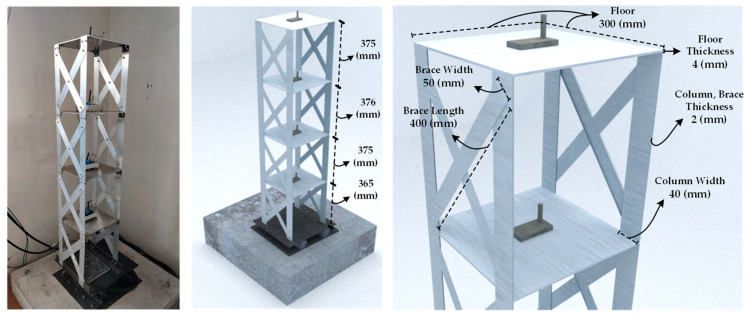
Four-story shear-type aluminum lab structure on the shaking table, along with dimensions.

**Figure 15:**
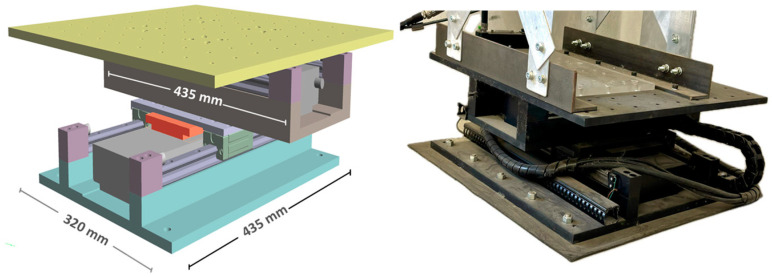
Picture and dimensions of the shaking table.

To validate the accuracy and functionality of the proposed system before its real-world application, an experimental campaign is conducted. Laboratory tests are carried out on a four-story shear-type aluminum structure placed on a shaking table, with Figure 14 depicting the structure with detailing its dimensions. As illustrated, each floor is equipped with a designed PCB, tasked with recording the structural vibrations induced by the ambient excitation for OMA. Furthermore, Figure 15 depicts the shaking table along with its dimensions, which is additionally employed to validate the results of the acceleration to displacement conversion, a topic to be further discussed in subsequent sections of this paper.

Multiple dynamic tests were conducted encompassing varied sampling rates, time intervals, and sampling durations. These experiments were undertaken without the imposition of external forces. Instead, the environmental vibrations generated by adjacent factory machinery were employed to simulate natural excitations acting upon the structure.

#### 4.3.1. Dynamic Verification against Shaking Table

This section aimed at the dynamic verification of the reliability and accuracy of the proposed system by comparing the resulting displacement from acceleration conversion of the sensor data via the one imposed by the shaking table.

Previous studies have proposed various methods for calculating displacement values from acceleration. These methods typically involve double integration of the acceleration data. However, relying solely on double integration may lead to unreliable results due to issues such as noise amplification, sensitivity to initial conditions, baseline drift, and numerical errors [39]. Lee [40] addressed these challenges by formulating the displacement reconstruction problem as a boundary value problem using second-order central finite differences. To improve accuracy, a finite-time interval was utilized to minimize errors between measured and approximated accelerations. Additionally, Park et al. [41] introduced an overlapping time-window technique to overcome limitations associated with non-overlapping time-windows, particularly in detecting abrupt structural damage.

**Figure 16:**
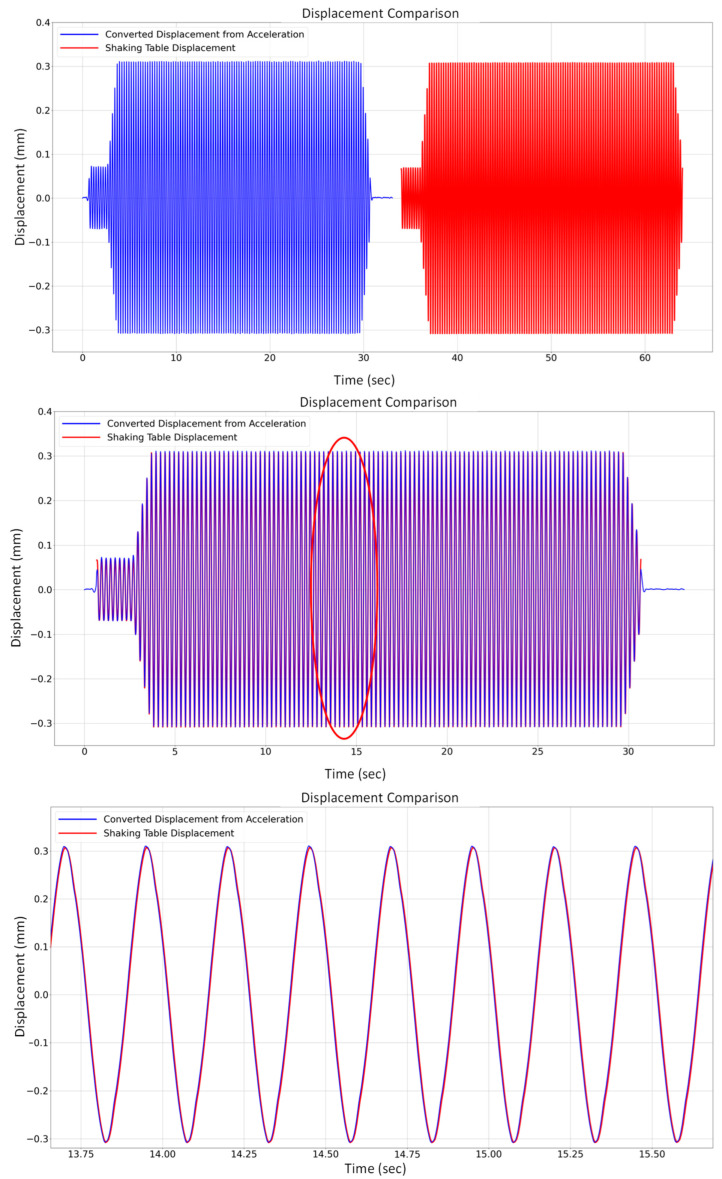
Comparison between the displacement obtained from the shaking table and the displacement derived from acceleration conversion.

In this paper, the displacement estimation is conducted using the acceleration data following the method proposed by Park et al. [41]. For this objective, an accelerometer was positioned on the shaking table to capture the acceleration data (in $m/s^{2}$) corresponding to the induced displacement (in mm). The accelerometer records the data at a sampling rate of 500 Hz. Concurrently, the imposed displacement (in the “*Y*” direction) by the shaking table is recorded using a connected PC to validate the converted displacement. This imposed displacement, applied at 4 Hz (well higher than the structure’s first natural frequency), has an amplitude of 20 mg and is expected to induce a maximum displacement of around 0.3 mm, as indicated by the displacement graph from the shaking table. As there is a sampling rate disparity between the recorded data by the shaking table (at 200 Hz) and the accelerometer (at 500 Hz), the data with the higher frequency rate are resampled to match the lower one for ease of comparison. As illustrated in Figure 16, a comparative illustration reveals substantial concurrence between the resultant displacement values resulting from the conversion of acceleration and those applied by the shaking table when compared graphically.

Following the estimation, a quantitative assessment was undertaken to discern the deviations between these respective plots. The mean absolute error (MAE) value is determined to be 0.018 mm, and the root mean squared error (RMSE) amounts to 0.023 mm. The discernibly low magnitudes of MAE and RMSE collectively signify a commendable degree of precision in the converted displacement inferred from the acceleration dataset.

**Figure 17:**
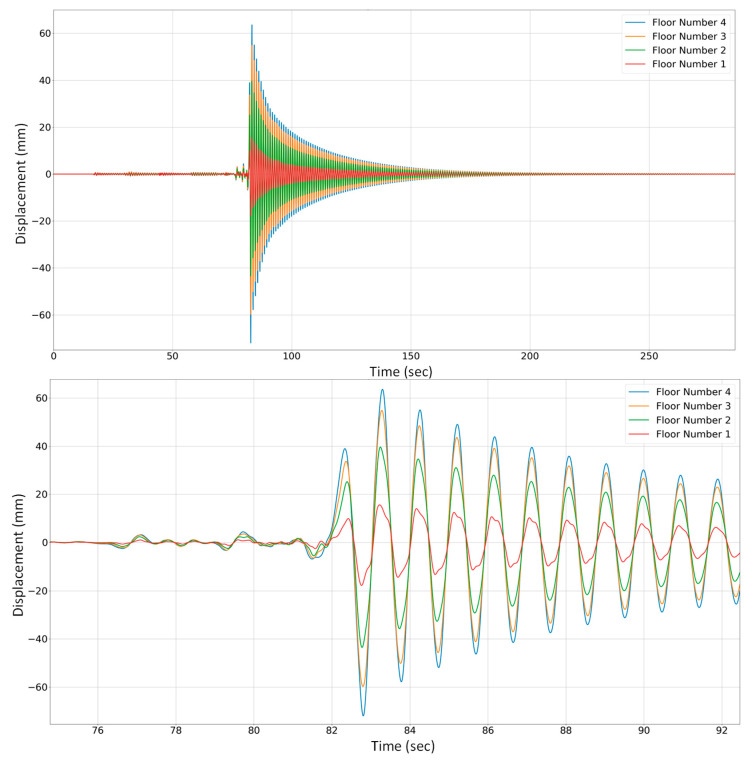
Displacement obtained from acceleration data for four floors.

After validating the precision of the displacement estimation, an experimental trial was conducted to evaluate the system’s effectiveness in calculating the displacements across different levels of the shear-type structure. It involved the placement of four accelerometers on each floor to capture the respective floor’s acceleration and a displacement of approximately 7 cm was then applied to the fourth floor toward the “*Y*” axis. The resulting maximum acceleration amplitude induced by this displacement was recorded at 1.74 g on the fourth floor. The converted displacements estimated through the acceleration-to-displacement conversion are depicted in Figure 17 for all four stories (in mm).

#### 4.3.2. Modal Identification Step

**Figure 18:**
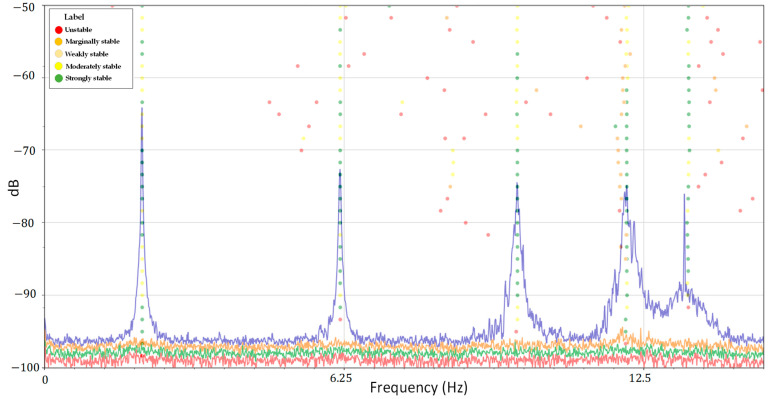
PSD and stabilization diagram of FDD and SSI method.

In the experimental test setup, the analytical phase involved the application of two modal identification methodologies, spanning both frequency and time domain frameworks: frequency domain decomposition (FDD) and stochastic subspace identification (SSI). Figure 18 illustrates the singular value decomposition (SVD) plot of the PSD obtained from the FDD method and the stabilization diagram from the SSI. This analysis was performed over a 30-min test duration, under ambient excitation conditions, using a frequency rate of 125 Hz, a resolution of 0.01, 50% overlapping, and the “Hann” windowing technique.

**Figure 19:**
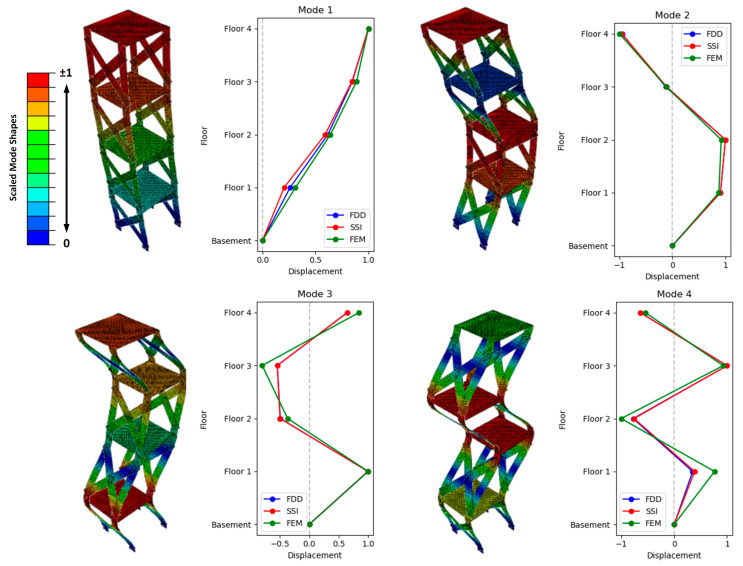
Comparison of the mode shapes, calculated by FDD, SSI, and FEM.

Table 4 presents a comparison of the extracted natural frequencies using PP, FDD, and SSI techniques, alongside those calculated via the FEM model. Additionally, it includes the computed damping ratios through SSI. Moreover, Figure 19 provides a visual representation of this comparison for the mode shapes.

**Figure 20:**
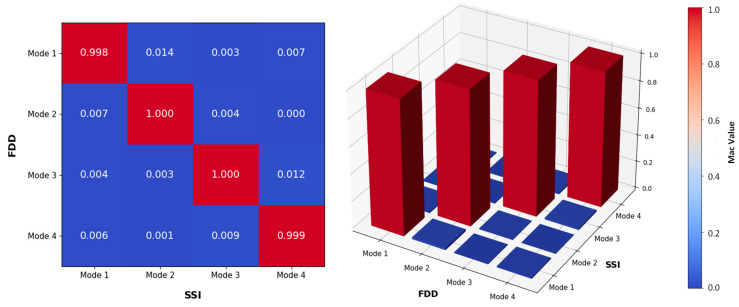
Comparison of the mode shapes calculated via FDD and SSI, reported by 2D and 3D MAC.

In order to have a quantitative measure to assess the similarity or correlation between mode shapes obtained from different methods, Figure 20 depicts the MAC values employed for the results derived from both FDD and SSI. The diagram illustrates diagonal elements close to 1, while non-diagonal elements approach zero, indicating substantial concurrence between mode shapes computed through time and frequency domain techniques. Additionally, it highlights significant orthogonality among the estimated mode shapes, suggesting that each mode shape represents a unique and clearly delineated spatial configuration of structural oscillation, distinct from those exhibited by other mode shapes within the system.

### 4.4. Real Case Test

This section focuses on the real-world application of the wireless monitoring system. It contains the procedures outlined in preceding sections for assessing the dynamic behavior of bridges, using modal identification methods such as PP, FDD, and EFDD in the frequency domain and SSI-Cov in the time domain. The computed modal parameters include natural frequencies, mode shapes, and damping ratios, all of which are subsequently reported.

#### 4.4.1. Bridge Description

**Figure 21:**
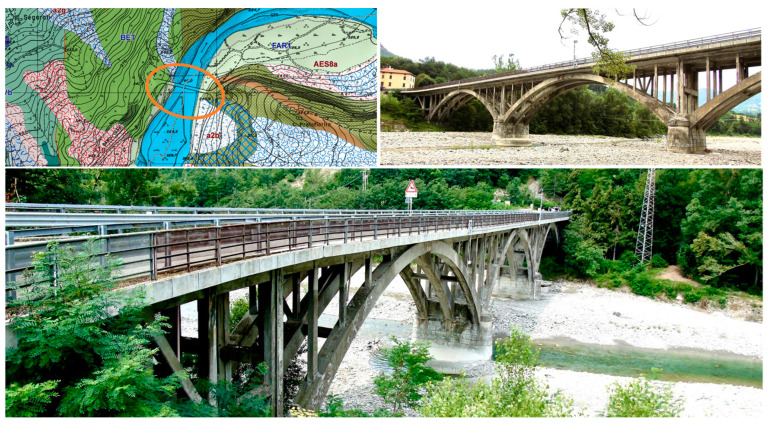
The Lamberti Bridge.

**Figure 22:**
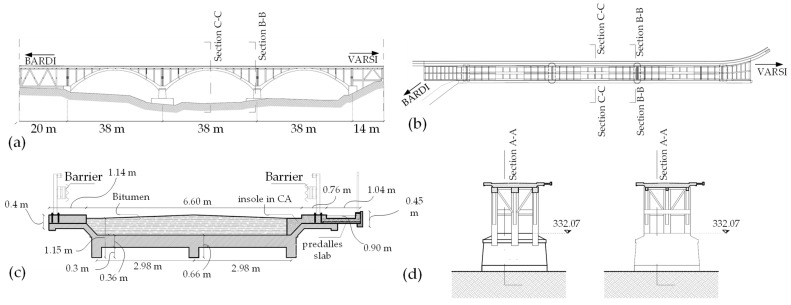
The geometric properties and cross-sectional details of the bridge: (**a**) longitudinal profile, (**b**) top view, (**c**) transverse cross-section, and (**d**) cross-section of the piers.

The selected real-world structure is the Lamberti Bridge, located in the province of Parma, Italy (situated at approximately 44.6509° N latitude and 9.8136° E longitude). Constructed in 1933, the bridge is built using reinforced concrete and is composed of three spans, each spanning 38 m. The bridge’s design features three interlinked arches connected to the deck. The deck comprises an arrangement of perpendicular beams that support a concrete slab of approximately 50 cm thick. Notably, the arch structures are interconnected by transverse beams positioned beneath the deck level. Figure 21 depicts the geographical positioning and visual representations of the Lamberti Bridge. Figure 22 also presents the geometric properties and cross-sectional details of the bridge.

#### 4.4.2. Data Acquisition

##### System Setup

**Figure 23:**
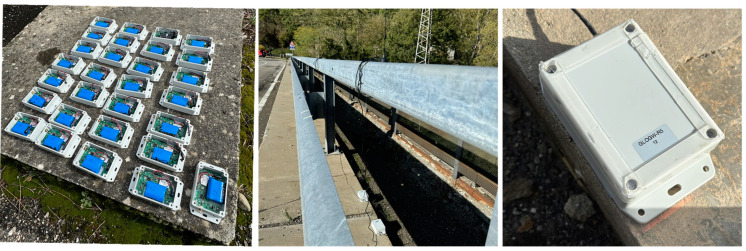
PCB containing MEMS accelerometers in the protective box.

**Figure 24:**
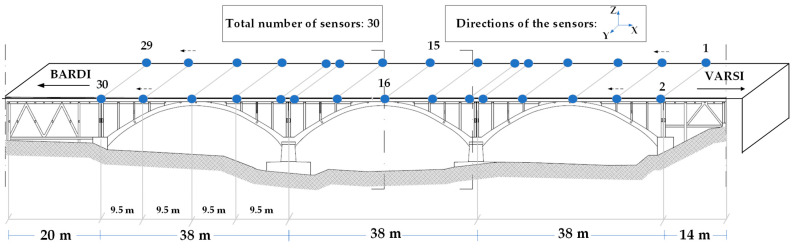
Test setup with 30 wireless sensors.

Each sensor was placed inside a protective box to ensure the precision of the measurements by minimizing the impact of environmental factors (see Figure 23). The operational vibration tests were carried out using 30 battery-powered, tri-axial MEMS accelerometers (*X*, *Y*, and *Z*), positioned in a single setup on the bridge (Figure 24) to capture the structural vibration simultaneously. The sensors were situated on both sides of the road, with odd-numbered sensors on the right side from Varsi to Bardi, and even-numbered sensors on the left. Each box was glued to the asphalt using bituminous adhesive tape in combination with regular double-sided tape; the bituminous tape is used for effective and long-lasting bonding to the asphalt, while the double-sided tape allows the box to be attached to the tape itself. An omnidirectional high-gain antenna (8 dBi) with a coaxial extension cable and magnetic base was selected. This allowed us to easily place the antennas above the guardrail in order to make communication easier and more reliable.

The gateway has been positioned exactly in the center of the bridge, on the right side from the Varsi to Bardi. The distance between it and the farthest sensor is thus just under 60 m, ensuring optimal communication without data loss. The RSSI value ranges between −45 dB for the boards closest to the gateway and −70 dB for the farthest ones.

**Figure 25:**
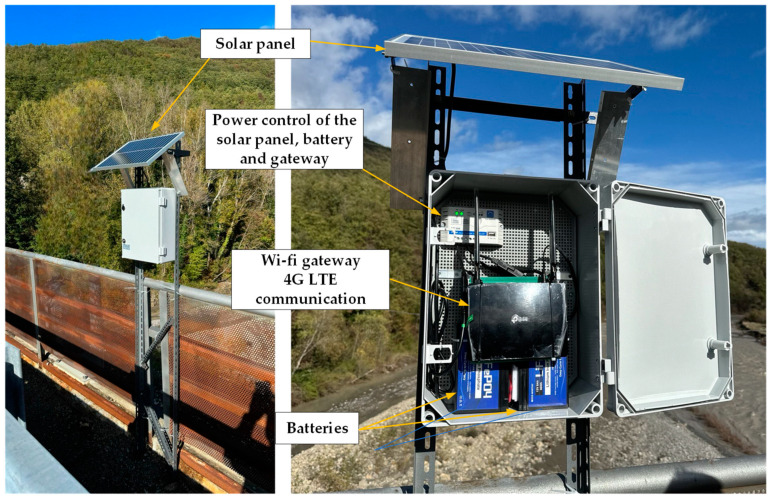
Wi-Fi gateway equipped with solar panel.

As depicted in Figure 25, to power the gateway, a 96 Wh battery pack consisting of two rechargeable 12 V lithium batteries was used. These batteries are in turn powered by a 25 W solar panel. The use of this configuration allowed the system to remain operational for several months.

##### System Configuration

The system was configured remotely via the web server to perform daily samplings of variable durations (from 30 to 120 min), with different frequencies (62.5, 125, or 250 Hz), and at varying times of the day. This last feature allowed for dynamic analysis of the structure under different temperatures and traffic conditions.

For the sake of simplicity and conciseness, this paper focuses solely on one of the tests and its respective results.

##### Challenges

After over four months of extended use of the system, it can be confirmed that the main challenge the system faces is related to the weather. In particular, prolonged periods of absence of sunlight lead to the depletion of the gateway batteries, resulting in a complete shutdown of the system. A similar situation occurs in case of issues with the 4G network.

However, the system still exhibits resilience; once the communication of the gateway with the network is restored, the monitoring resumes.

#### 4.4.3. Operational Modal Analysis

In order to identify the experimental modes of the bridge, vibration measurements were performed on the structure under operational conditions. The excitation of the structure was mainly ambient, due to mild wind and traffic on both lanes. The tests were performed during the daytime and the traffic was not very busy, but there was generally at least one vehicle passing over the tested bridge for each 5 min. The passing vehicles included cars and some heavy trucks.

##### Pre-Processing Step

**Figure 26:**
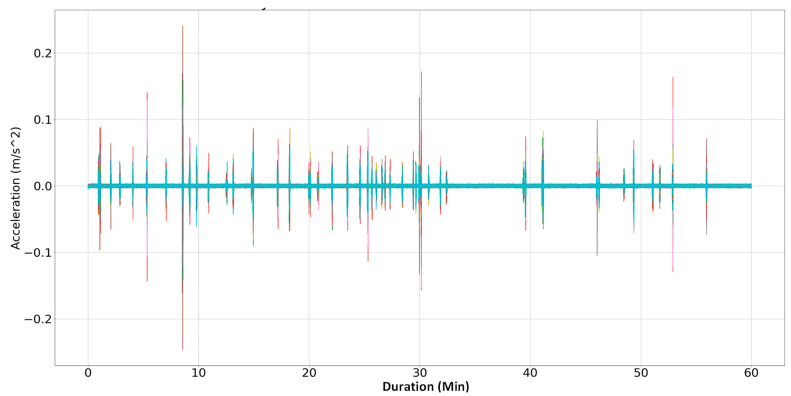
Pre-processing results for all sensor recordings in Z direction.

The data collected from 30 tri-axial sensors underwent pre-processing for synchronization and trend removal, following the mentioned method in Section 3. Additionally, a low-pass filter, Butterworth type, with a cutoff frequency of 15 Hz and the order of 2 was applied to mitigate potential unwanted noises in the raw data. Figure 26 illustrates the filtered, synchronized, detrended acceleration data from all sensors along the *Z*-axis.

##### Modal Identification Methods

**Figure 27:**
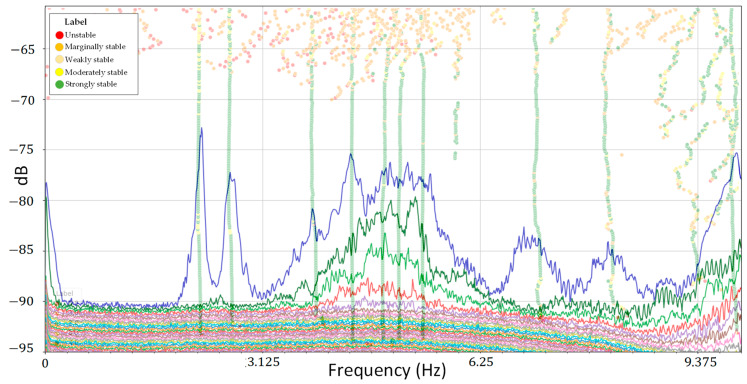
Combination of the PSD with stabilization diagram.

To determine the modal characteristics of the bridge using pre-processed data, four techniques were employed: PP, FDD, and EFDD in the frequency and SSI-Cov in the time domains. Figure 27 shows the SVD of the PSD derived from the frequency domain methods, along with the stabilization diagram of SSI. The SVD plot used 0.01 as the resolution, 50% overlapping, and a “Hann” window, and the stabilization diagram used 15 as the number of block rows (time lags), with an order max of 1000.

Table 5 provides the comparison of the estimated natural frequencies using PP, FDD, EFDD, and SSI methodologies. Additionally, it presents the calculated damping ratios obtained using the SSI approach.

Table 6 presents a comparative analysis between the natural frequencies determined through modal identification methods in the presented study and those documented in a thesis published in 2018 [42], which employed the SSI method to determine them of the Lamberti Bridge. It is noteworthy that the two tests were carried out in the same month of the year with five years’ differences. In the mentioned thesis, 18 designated positions were specified for placing wired sensors on the bridge in different setups. In contrast, this paper employed 30 tri-axial wireless sensors, resulting in a relatively larger number of calculated natural frequencies. The table juxtaposes these natural frequencies reported in the thesis with their corresponding natural frequencies in the present study for comprehensive evaluation.

As outlined in Table 6, comparing the derived natural frequencies with those from 2018 indicates reductions in the first three modes by 2.43%, 14.80%, and 7.89%, respectively. However, it is important to acknowledge the challenges inherent in comparing the dynamic characteristics of structures, particularly when assessing the structural health of bridges. Distinguishing between normal and abnormal changes in dynamic properties over time is crucial. Changes typically stem from deviations in material properties, indicating potential damage, such as stiffness loss. Conversely, normal variations are primarily influenced by environmental and operational factors. External conditions such as temperature, humidity, and traffic loads can lead to stiffness variations, subsequently impacting bridge dynamics. Temperature fluctuations, for instance, cause daily frequency shifts of around 5% and seasonal shifts of 10%. Moreover, operational conditions contribute to over 5% of fluctuations [4]. However, Table 6 highlights disparities in these frequencies compared to data from five years ago, at the same time of the year under identical weather and temperature conditions. Nevertheless, an investigation is necessary to understand the underlying modifications in their values, such an inquiry lies outside the scope of the current study.

**Figure 28:**
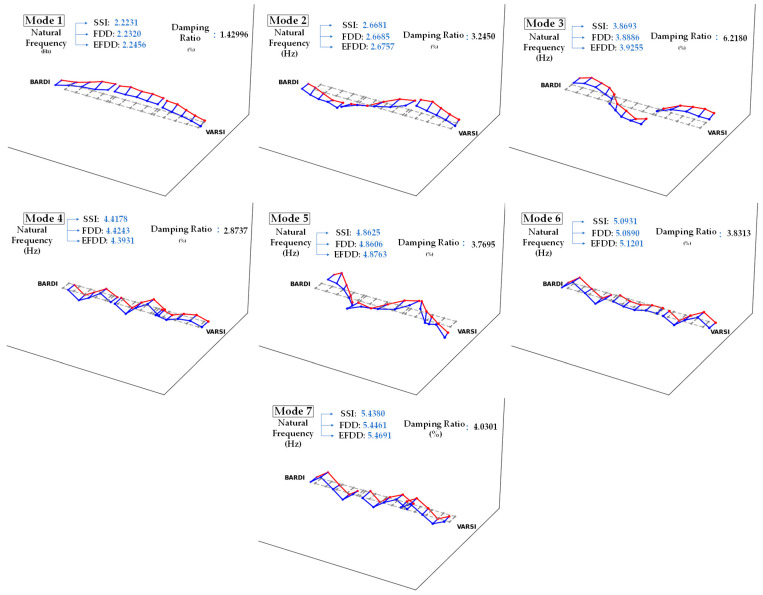
Extracted dynamic parameters of the Lamberti Bridge.

**Figure 29:**
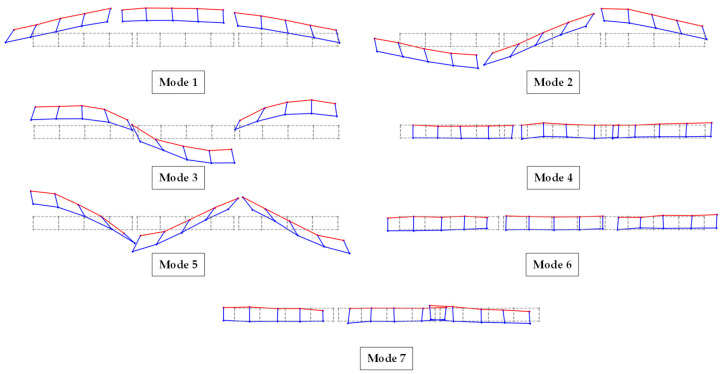
First seven mode shapes of the bridge in the XY plane.

In Figure 28, detailed results of the application of modal identification techniques to the Lamberti Bridge are presented. The figure showcases the initial seven mode shapes of the structure, along with their corresponding natural frequencies obtained through various modal identification techniques and the accompanying damping ratios. Figure 29 additionally illustrates the mode shapes of the bridge on the XY plane.

**Figure 30:**
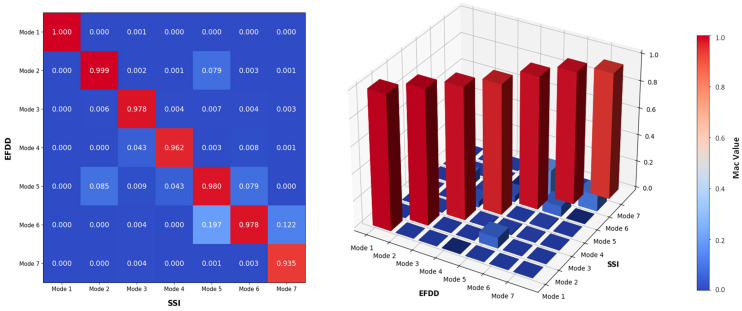
Comparison of the mode shapes of the bridge, calculated via EFDD and SSI, reported by 2D and 3D MAC.

To assess the correspondence between the calculated mode shapes derived from the EFDD and SSI-Cov, the 2D and 3D MAC plot using the collected dataset from the Lamberti Bridge is shown in Figure 30. This plot serves as a quantitative means to evaluate the similarity and the agreement between the mode shapes obtained through these techniques in different domains. As can be concluded from the MAC plot, there is a noticeable agreement between the calculated mode shapes derived from time and frequency domain analysis. It indicates a high level of concurrence and orthogonality among the first 7 estimated mode shapes. This suggests that each mode shape portrays a distinct and precisely defined spatial arrangement of the bridge vibration, separate from the characteristics exhibited by other mode shapes within the system.

#### 4.4.4. Displacement Calculation, Using Acceleration Data

One approach for the determination of a bridge’s displacement is the direct assessment, which employs fixed reference-based technologies. Examples include linear variable differential transformers (LVDTs) [43], laser Doppler vibrometers (LDVs) [44], and vision-based systems [45,46,47]. While these measurement methods offer high accuracy, they are frequently deemed impractical due to their associated costs and installation complexities. Moreover, a notable drawback is their reliance on a fixed reference point, a requirement that is often unachievable in the context of full-scale civil structures [48].

An alternative to these methodologies involves the utilization of strain gauges or accelerometers, such as MEMS, as they eliminate the need for a stationary reference point, thereby enhancing operational feasibility. However, practical challenges arise with accelerometers facing difficulty in accurately measuring low-frequency components, and strain gauges struggling with precision due to susceptibility to measurement noise, particularly in the high-frequency range. To address these challenges, previous studies have introduced indirect displacement estimation, using the acceleration and strain (IDEAS) method [49,50]. 

Notably, installing an accelerometer is less time-consuming compared to a strain gauge, making the application of accelerometers more practical. However, concerns arise in their application, as data include errors stemming from limitations in the analog-to-digital conversion process and sensor noise. Mitigating them proves challenging due to the random nature of live loads on the bridge, with forced displacement frequencies. Beyond measurement errors, obtaining accurate initial and terminal conditions for numerical double integration is hindered by continuous vibrations induced by live load conditions. Consequently, precise displacement calculation becomes an important task.

Given the intricate nature of bridge dynamics, it is imperative to acknowledge that the response to live loads predominantly manifests at lower frequencies, typically below 1 Hz [35]. This dependency on frequency is notably influenced by the length of the bridge under consideration, as well as its fundamental frequencies. This highlights the importance of accurately evaluating force–displacement relationships within this frequency band. Expanding on this idea, Sekiya et al. [35] introduced the “free vibration separation method.” This method involves setting initial and final displacement conditions and assuming sinusoidal oscillations around the zero-axis at the bridge’s free vibration frequency. These oscillations cover both fundamental and higher-order-mode vibration frequencies, both before and after a vehicle passes. The process involves calculating the displacement of free vibration and then numerically integrating the acceleration related to the forced displacement component during the vehicle’s passage. The total displacement, comprising both the free vibration and estimated forced displacement components, is then determined by summation. This method offers a comprehensive framework for understanding and quantifying how bridges dynamically respond to live loads within the specified frequency range.

Nevertheless, this paper incorporates the mentioned method with the integration of a Kalman filter to effectively tackle challenges related to noise, dynamic behavior, and the imperative for adaptive estimation displacement data out of acceleration. The Kalman filter, a set of mathematical equations, estimates the state of a process by minimizing the average square error. It possesses the advantage of estimating a situation based on minimal data. The Kalman filter equation consists of two groups: the time update equation (predicted process) and the measurement update equation (correct process). The time update uses a state estimate from a previous time to predict the current state, while the measurement update incorporates current measurement information to enhance predictions and achieve a more accurate state estimate [51]. Below are the equations for predictions and corrections in the Kalman filter [51]:

Prediction:

(1) $${\hat{X}}_{t\left|t-1\right.}=F_{t}{\hat{X}}_{t-1\left|t-1\right.}+B_{t}U_{t}$$

(2) $$P_{t\left|t-1\right.}=F_{t}P_{t-1\left|t-1\right.}F_{t}^{T}+Q_{t}$$

Correction:

(3) $${\hat{X}}_{t\left|t\right.}={\hat{X}}_{t\left|t-1\right.}+K_{t}(y_{t}-H_{t}{\hat{X}}_{t\left|t-1\right.})$$

(4) $$K_{t}=P_{t\left|t-1\right.}H_{t}^{T}{\left(H_{t}P_{t\left|t-1\right.}H_{t}^{T}+R_{t}\right)}^{-1}$$

(5) $$P_{t\left|t\right.}=(I-K_{t}H_{t})P_{t\left|t-1\right.}$$

Here, $\hat{X}$ represents the estimated state, F is the state transition matrix, U is a control variable, B is the control matrix, H is the measurement matrix, y is the measurement variable, T is the process variance matrix, P is the state variance matrix, K is the Kalman filter gain, Q is the process variance matrix, R is the measurement variance matrix, and t is the time step.

**Figure 31:**
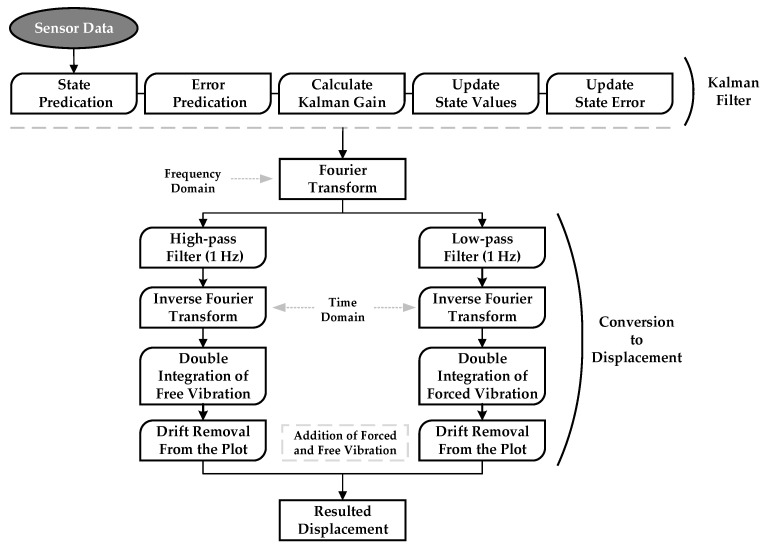
Flowchart of the proposed method for displacement estimation.

Figure 31 is the flowchart of the proposed method, which is the integration of the free vibration separation method and the implementation of the Kalman filter.

As depicted in Figure 31, acceleration data first undergo a Kalman filter involving several key steps. Initially, the filter predicts the current state of the system using a dynamics model and the previous state. Simultaneously, it estimates the associated state prediction error, considering uncertainties in the initial state and system dynamics. The Kalman gain is then calculated to determine the influence of the prediction and measurement on the final state update. The predicted state is adjusted based on the actual measurement, with the Kalman gain determining the weight assigned to each source of information. Subsequently, the filter refines its estimate of the state error by combining the predicted error and measurement error. This iterative process enhances the accuracy of the state estimates by iteratively reconciling predictions and measurements while accounting for uncertainties in the system.

**Figure 32:**
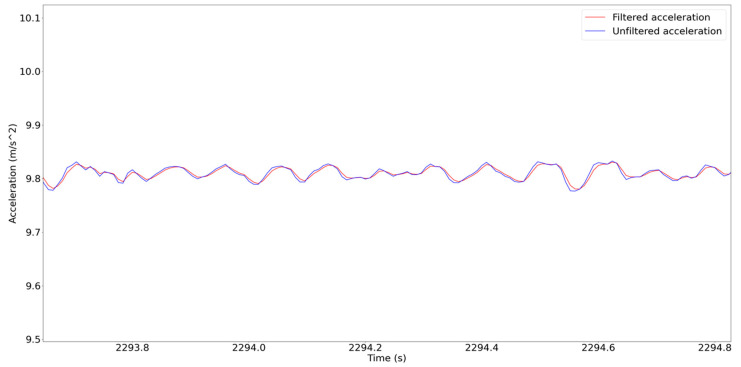
Comparison between the acceleration data before and after the implementation of the Kalman filter.

Illustrated in Figure 32 is a comparison between the acceleration data before and after the application of the Kalman filter. The data utilized for analysis comprised the recorded acceleration data from sensor number 16 in the Z direction. This sensor was positioned at the midpoint of the middle span, capturing readings following the passage of a truck over the bridge. Notably, the MAE and RMSE values between the filtered and unfiltered acceleration are 0.039 m/s^2^ and 0.090 m/s^2^, respectively. These values signify a reduction of the noises in the recorded data.

**Figure 33:**
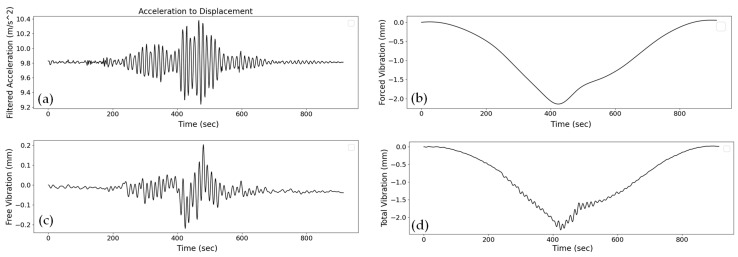
Acceleration conversion to the displacement from sensor number 16 in the Z-direction by the proposed method: (**a**) acceleration data after applying the Kalman filter, (**b**) forced vibration plot, (**c**) free vibration data, and (**d**) resulting total displacement of the bridge.

As mentioned in the flowchart in Figure 31, after the application of the Kalman filter, to calculate the forced and free displacement of the bridge after the truck crossing, the process involves separating free and forced vibration regions based on vehicle entry and exit detection. The acceleration data are then transformed from the time domain to the frequency domain using Fourier transform. Frequencies below and above 1.0 Hz are removed to isolate free vibration and forced displacement effects, respectively. The accelerations in the time domain are estimated by taking the inverse Fourier transform. To obtain displacement during free vibration, the acceleration is numerically integrated twice from the point where it is zero before the vehicle enters until it returns to zero after the vehicle exits. A drift component is subtracted to align initial and terminal displacements with zero. Similarly, for forced displacement, acceleration is integrated twice from the vehicle entry to the exit. Again, a drift component is subtracted to ensure zero initial and terminal displacements. The total displacement, incorporating both free and forced vibration components, is then obtained by linearly summing the displacements. The result is depicted in Figure 33.

## 5. Conclusions

This paper demonstrates the effectiveness of the discussed wireless DAQ system for OMA of bridges and showcases its potential for real-world applications. It employs costume-designed wireless sensors powered by batteries and features 3D MEMS accelerometers, the ADXL355 version. These sensors are controlled by a microcontroller that provides dynamic voltage scaling to optimize power consumption while maintaining high processing capabilities. Additionally, it incorporates a simple, low-power, and cost-effective ESP32 Wi-Fi module. To enhance energy efficiency, the system employs hardware components with low power consumption and features a standby mode, thereby reducing continuous power usage. This design ensures the system’s uninterrupted operation for a minimum of five years. This study also addresses the common challenges encountered in the implementation of wireless communication.

As stated in the paper, the validation and the comparison of the dynamic parameters derived from the Lamberti Bridge are compared with data from five years ago under identical weather and temperature conditions. Nevertheless, it is crucial to acknowledge that environmental and operational factors, such as temperature fluctuations and humidity variations, directly and inevitably influence these parameters. The deviations resulting from these factors should not be misconstrued as structural damage, and false alarms in the monitoring system should be avoided. To mitigate the dominating influence of these variables, forthcoming efforts will prioritize integrating these effects within the analytical process and also incorporating AI methods to model dynamic behavior, distinguishing structural variations, such as stiffness, from external influences. Additionally, enhancements to modal identification algorithms in both the time and frequency domains, as well as advancements in signal processing methods, are under consideration. These improvements aim to enhance result precision while concurrently reducing computational expenses. It is also conceivable to develop a user-friendly graphical interface that encompasses the entire data analysis process, spanning from preliminary data processing to subsequent stages, for applications in wireless monitoring systems.

## Figures and Tables

**Table 1:** Key parameters of the ADXL355 version [37].

Attribute	Testing Conditions/Remarks	Average Value	Measurement Unit
Sensitivity	±2 g (X, Y, and Z)	256,000	LSB/g
Noise (spectral density)	±2 g (X, Y, and Z)	22.5	$\mu g/\sqrt{Hz}$
Sensitivity change due to temperature	−40 °C to+125 °C	±0.01	%/°C
Nonlinearity	±2 g	0.1	%
Zero offset	±2 g (X, Y, and Z)	25	mg

**Table 2:** Average power consumption of the system.

Stages	Power Consumption	Time Per Day
Transmission	125 mA	20 s
Sampling	3 mA	30 min
Stand-by	5.5 uA	24 h

**Table 3:** Sensor noise characteristics across three axes.

Noise Axis	Peak to Peak (mg)	RMS Noise (mg)	Noise Density $(\mu g/\sqrt{Hz}$)
X	0.6899	0.0891	17.79
Y	1.0250	0.0909	19.57
Z	1.0173	0.1191	22.49
Average	0.9108	0.0997	19.95

**Table 4:** Comparison of the natural frequencies calculated by PP, FDD, and SSI methods.

Modes	Natural Frequency (Hz)				Relative Difference (%)				Damping Ratio (%)
	PP	FDD	SSI	FEM	PP vs. SSI	FDD vs. SSI	FEM vs. FDD	FEM vs. SSI	SSI
First	2.03	2.03	2.03	2.07	<0.01	<0.01	1.98	2.01	1.08
Second	6.15	6.15	6.14	6.14	0.16	0.32	0.02	0.04	0.28
Third	9.81	9.8	9.81	9.96	<0.01	0.2	1.52	2.61	0.35
Fourth	12.15	12.15	12.18	12.69	0.25	0.48	4.1	6.07	0.53

**Table 5:** Comparison of the calculated natural frequencies and estimated damping ratios for each mode.

Modes	Type	Natural Frequency (Hz)				Relative Difference (%)			Damping (%)
		PP	FDD	EFDD	SSI_Cov	FDD vs. EFDD	FDD vs. SSI_Cov	EFDD vs. SSI_Cov	SSI
First	First transverse	2.2	2.23	2.25	2.22	−0.89	0.4	1	1.43
Second	Second transverse	2.7	2.67	2.68	2.67	−0.27	0.01	0.28	3.24
Third	Third transverse	3.9	3.89	3.93	3.87	−0.95	0.5	1.43	6.22
Fourth	First vertical	4.4	4.42	4.39	4.42	0.71	0.15	−0.56	2.87
Fifth	First torsion	4.9	4.86	4.88	4.86	−0.32	−0.04	0.28	3.77
Sixth	Second vertical	5.1	5.09	5.12	5.09	−0.61	−0.08	0.53	3.83
Seventh	Third vertical	5.4	5.45	5.47	5.44	−0.42	0.15	0.57	4.03

**Table 6:** Comparison of the calculated natural frequencies with 2018.

Modes	Natural Frequency (Hz)			Relative Difference (%)		
	Thesis [42]	EFDD	SSI-Cov	Thesis vs. EFDD	Thesis vs. SSI-Cov	Average
First	2.29	2.25	2.22	−1.75	−3.11	−2.43
Second	3.10	2.68	2.67	−13.55	−16.04	−14.80
Third	4.22	3.93	3.87	−6.87	−8.91	−7.89

## Data Availability

The data presented in this study are available on request from the corresponding author.
